# Supplementary figures and images for: The association between exposure to interferon-beta during pregnancy and birth measurements in offspring of women with multiple sclerosis
Source: PLoS One. 2019 Dec 30;14(12):e0227120. doi: 10.1371/journal.pone.0227120 (PMC6936848; doi:10.1371/journal.pone.0227120)

**S2 Figure**- Birth height in cms by gestational age, according to interferon-beta exposure status


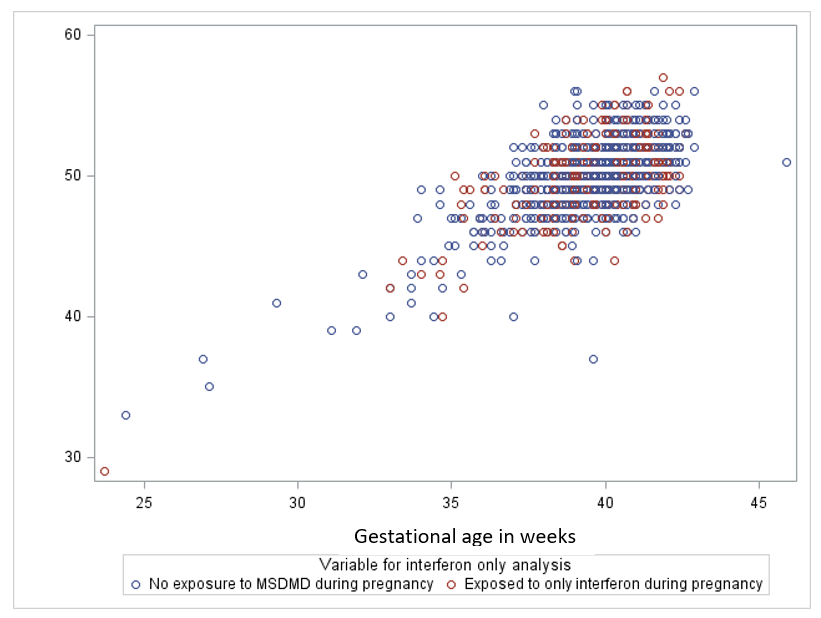

Supplement: S2 Fig — (DOCX) [file pone.0227120.s002.docx]

**S3 Figure**- Head circumference by gestational age, according to interferon-beta exposure status


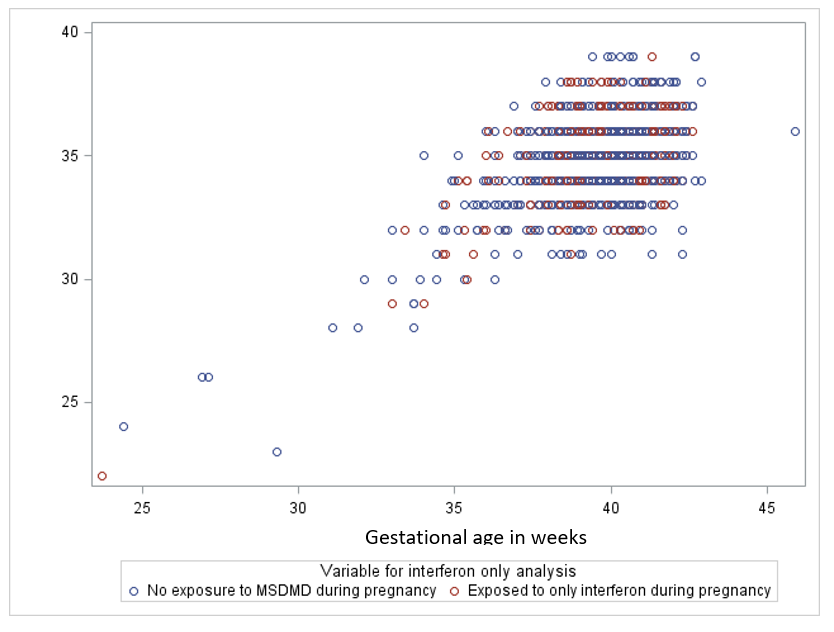

Supplement: S3 Fig — (DOCX) [file pone.0227120.s003.docx]
