# Supplementary material for: The association between exposure to interferon-beta during pregnancy and birth measurements in offspring of women with multiple sclerosis
Source: PLoS One. 2019 Dec 30;14(12):e0227120. doi: 10.1371/journal.pone.0227120 (PMC6936848; doi:10.1371/journal.pone.0227120)
Supplement: S1 Table — (DOCX) [file pone.0227120.s004.docx]

**S1 Table**- ATC codes and brand names used to identify interferon-beta exposure

| **Treatments used to identify interferon exposure** | | |
| --- | --- | --- |
| **Brand names** | | Avonex Plegidry Betaferon Extavia Rebif |
| **ATC codes** | | L03AB07 L03AB08 L03AB13 |
| **Treatments used to identify any MSDMD (same as used to identify interferon-beta plus the codes listed below)** | | |
| **Brand names** | Copaxone Gilenya IVIG Tecfidera Mitoxantrone Aubagio Lemtrada Tysabri Cladribine | |
| **ATC codes** | J06BA02 L01AA01 L01BA01 L04AX03 L01BB04 L01DB07 L01XC04 L03AX13 L04AA13 L04AA23 L04AA27 L04AA31 L04AX01 N07XX09 | |
