# Supplementary material for: The association between exposure to interferon-beta during pregnancy and birth measurements in offspring of women with multiple sclerosis
Source: PLoS One. 2019 Dec 30;14(12):e0227120. doi: 10.1371/journal.pone.0227120 (PMC6936848; doi:10.1371/journal.pone.0227120)
Supplement: S2 Table — (DOCX) [file pone.0227120.s005.docx]

**S2 Table**- Exposed to interferon-beta according to all possibly exposed sensitivity analysis (Sweden only)

|  |  | **Mean(SE)** | **Mean(SE)** | **Mean(SE)** | **Mean(SE)** |
| --- | --- | --- | --- | --- | --- |
|  | **N** | **Gestational age in weeks** | **Birth weight in grams** | **Birth height in cm's** | **Head circumference in cm** |
| **Exposed to IFN-beta** | 447 | 39.7 (0.1) | 3463.1 (26.3) | 50.1 (0.1) | 35.0 (0.1) |
| **Not exposed to MSDMD** | 611 | 39.5 (0.1) | 3427 (23.5) | 50.0 (0.1) | 34.7 (0.1) |
| **Differently exposed siblings** |  |  |  |  |  |
| **Exposed sibling** | 41 | 39.9 (0.2) | 3512.4 (76.0) | 50.3 (0.4) | 35.0 (0.2) |
| **Unexposed sibling** | 41 | 39.1 (0.3) | 3303.6 (100.8) | 49.4 (0.5) | 34.5 (0.3) |
| *Some are no longer counted as unexposed to any MSDMD- identified using MS reg for other MSDMD treatments | | | | | |
