# Supplementary material for: The association between exposure to interferon-beta during pregnancy and birth measurements in offspring of women with multiple sclerosis
Source: PLoS One. 2019 Dec 30;14(12):e0227120. doi: 10.1371/journal.pone.0227120 (PMC6936848; doi:10.1371/journal.pone.0227120)
Supplement: S3 Table — (DOCX) [file pone.0227120.s006.docx]

**S3 Table**- Exposure to any MSDMD’s sensitivity analysis

|  |  | **Mean(SE)** | **Mean(SE)** | **Mean(SE)** | **Mean(SE)** |
| --- | --- | --- | --- | --- | --- |
|  | **N** | **Gestational age in weeks** | **Birth weight in grams** | **Birth height in cm's** | **Head circumference in cm** |
| **Sweden** |  |  |  |  |  |
|  |  |  |  |  |  |
| **Exposed to MSDMD** | 632 | 39.7 (0.1) | 3463.4 (22.8) | 50.1 (0.1) | 34.9 (0.1) |
| **Not exposed to MSDMD** | 835 | 39.5 (0.1) | 3414.8 (19.4) | 50.0 (0.1) | 34.8 (0.1) |
| **Differently exposed siblings** |  |  |  |  |  |
| **Exposed sibling** | 87 | 39.8 (0.2) | 3411.9 (51.1) | 50.0 (0.3) | 34.9 (0.2) |
| **Unexposed sibling** | 85 | 39.4 (0.2) | 3347.8 (59.6) | 49.7 (0.3) | 34.7 (0.2) |
| **Finland** |  |  |  |  |  |
|  |  |  |  |  |  |
| **Exposed to MSDMD** | 315 | 39.6 (2.2) | 3389.6 (589.9) | 49.6 (2.9) | 34.6 (2.0) |
| **Not exposed to MSDMD** | 331 | 39.5 (1.9) | 3410.4 (541.0 | 49.6 (2.5) | 34.8 (1.7) |
| **Differently exposed siblings** |  |  |  |  |  |
| **Exposed sibling** | 59 | 39.4 (2.6) | 3347.1 (571.3) | 49.3 (3.3) | 34.5 (2.3) |
| **Unexposed sibling** | 59 | 39.8 (1.2) | 3509.5 (411.1) | 50.0 (1.9) | 35.0 (1.4) |
