# Supplementary material for: The association between exposure to interferon-beta during pregnancy and birth measurements in offspring of women with multiple sclerosis
Source: PLoS One. 2019 Dec 30;14(12):e0227120. doi: 10.1371/journal.pone.0227120 (PMC6936848; doi:10.1371/journal.pone.0227120)
Supplement: S4 Table — (DOCX) [file pone.0227120.s007.docx]

**S4 Table-** GEE OLS unadjusted models

|  | **Unadjusted** | |  |  |  |  |
| --- | --- | --- | --- | --- | --- | --- |
|  | **Weight** |  | **Height** |  | **Head circumference** | |
|  | **Beta (SE)** | **P-value** | **Beta (SE)** | **P-value** | **Beta (SE)** | **P-value** |
| **Sweden** |  |  |  |  |  |  |
|  |  |  |  |  |  |  |
| **Overall** | 48.5 (33.2) | 0.14 | 0.1 (0.1) | 0.56 | 0.2 (0.1) | 0.08 |
| **Differently exposed siblings** | 64.1 (77.3) | 0.41 | 0.3 (0.3) | 0.4 | 0.2 (0.2) | 0.47 |
| **Finland** |  |  |  |  |  |  |
|  |  |  |  |  |  |  |
| **Overall** | -20.8 (50.4) | 0.68 | 0.03 (0.2) | 0.91 | -0.16 (0.17) | 0.36 |
| **Differently exposed siblings** | -162.5 (92.1) | 0.08 | -0.68 (0.57) | 0.24 | -0.47 (0.40) | 0.23 |
