# Supplementary material for: The association between exposure to interferon-beta during pregnancy and birth measurements in offspring of women with multiple sclerosis
Source: PLoS One. 2019 Dec 30;14(12):e0227120. doi: 10.1371/journal.pone.0227120 (PMC6936848; doi:10.1371/journal.pone.0227120)
Supplement: S5 Table — (DOCX) [file pone.0227120.s008.docx]

**S5 Table**- GEE OLS adjusted models

|  | **Adjusted*** | |  |  |  |  |
| --- | --- | --- | --- | --- | --- | --- |
|  | **Weight** |  | **Height** |  | **Head circumference** | |
|  | **Beta (SE)** | **P-value** | **Beta (SE)** | **P-value** | **Beta (SE)** | **P-value** |
| **Sweden** |  |  |  |  |  |  |
|  |  |  |  |  |  |  |
| **Overall** | 33.6 (25.5) | 0.19 | 0.0 (0.1) | 0.91 | 0.1 (0.1) | 0.09 |
| **Differently exposed siblings** | -3.8 (53.3) | 0.94 | -0.04 (0.2) | 0.85 | 0.0 (0.2) | 0.96 |
| **Finland** |  |  |  |  |  |  |
|  |  |  |  |  |  |  |
| **Overall** | -25.0 (40.5) | 0.54 | 0.05 (0.2) | 0.8 | -0.14 (0.13) | 0.29 |
| **Differently exposed siblings** | -80.2 (58.1) | 0.17 | -0.13 (0.30) | 0.67 | -0.09 (0.2) | 0.66 |
| *Adjusted for gestational age, sex of the newborn, smoking status of the mother, and maternal age at LMP | | | | | | |
