# Supplementary material for: The association between exposure to interferon-beta during pregnancy and birth measurements in offspring of women with multiple sclerosis
Source: PLoS One. 2019 Dec 30;14(12):e0227120. doi: 10.1371/journal.pone.0227120 (PMC6936848; doi:10.1371/journal.pone.0227120)
Supplement: S6 Table — (DOCX) [file pone.0227120.s009.docx]

**S6 Table-** Exposure to any MSDMD’s all possibly exposed sensitivity analysis (Sweden only)

|  |  | **Mean(SE)** | **Mean(SE)** | **Mean(SE)** | **Mean(SE)** |
| --- | --- | --- | --- | --- | --- |
|  | **N** | **Gestational age in weeks** | **Birth weight in grams** | **Birth height in cm's** | **Head circumference in cm** |
| **Exposed to MSDMD** | 856 | 39.6 (0.1) | 3442.0 (19.0) | 50.0 (0.1) | 34.9 (0.1) |
| **Not exposed to MSDMD** | 611 | 39.5 (0.1) | 3427.0 (23.5) | 50.0 (0.1) | 34.8 (0.1) |
| **Differently exposed siblings** |  |  |  |  |  |
| **Exposed sibling** | 67 | 39.9 (0.2) | 3463.4 (55.6) | 50.2 (0.3) | 34.9 (0.2) |
| **Unexposed sibling** | 67 | 39.3 (0.2) | 3338.5 (71.1) | 49.6 (0.3) | 34.6 (0.2) |
| *More mothers could have been identified as using MSDMD's- so more cases where both pregnancies MSDMD exposed | | | | | |
