# Supplementary material for: The association between exposure to interferon-beta during pregnancy and birth measurements in offspring of women with multiple sclerosis
Source: PLoS One. 2019 Dec 30;14(12):e0227120. doi: 10.1371/journal.pone.0227120 (PMC6936848; doi:10.1371/journal.pone.0227120)
Supplement: Attachment — Submitted filename: Birth registry review.docx [file pone.0227120.s010.docx]

Birth registry –exposure to BIFN and all DMDs

This is a registry study, with limitationsinherent to that methodology, of women with MS in Sweden and, separately in Finland, who were exposed to BIFN and to other DMDS compared to MS women who delivered live births who were not.

1. Reviewer would like to see in Methods or elsewhere comments by the authors on the powering of the study. Although no significant difference in bodily measures of newborns were uncovered in either Sweden or Finland, there were trends in each country’s cohort. Can a statement be made regarding what degree of difference would these sized groups be empowered to detect?
2. This study examined the size of newborns. To be counted, a live birth must have occurred. One limitation not mentioned is termination of pregnancies (miscarriages or abortions) which may have occurred in these women. This should be mentioned. Is there any way to uncover the rate of selective or non-selective terminations and how that may have affected the study results?
3. The investigators identified sibling pairs of mothers with MS, in which one was exposed and the sibling was not exposed to BIFN, and found no differences in body measures of infant siblings based on exposures. It would also be of interest to know what the power for detecting a difference would be. For example, would it be powered sufficiently to detect a 10% difference in one of the measures? And, could the authors mention what degree of difference in head circumference or other measures is considered significant?
4. The study results are averages. Is there a way to identify the numbers of outliers in each group? Numbers/rates of premature infants?
5. The authors speculate that, due to size, beta-interferons will not cross the placental barrier due to its low permeability for high molecular weight substances. Are there not data (even from animal studies) to cite which can provide more definitive information on whether beta-interferons can cross the placenta, or not?
